# Supplementary material for: Surveillance of WHO Priority Gram-Negative Pathogenic Bacteria in Effluents from Two Seafood Processing Facilities in Tema, Ghana, 2021 and 2022: A Descriptive Study
Source: Int J Environ Res Public Health. 2022 Aug 30;19(17):10823. doi: 10.3390/ijerph191710823 (PMC9518130; doi:10.3390/ijerph191710823)
Supplement: Supplementary file 1 [file ijerph-19-10823-s001.zip › ijerph-1873579 Supplementary/ijerph-1873579 Supplementary data.pdf]

Supplementary Data

Table S1. Physical characteristics of effluent samples in two seafood processing facilities (SPF) in Tema, Ghana during 2021 and 2022

Date of sample collection	pH		Temperature (°C)	
	**SPF-1**	**SPF-2**	**SPF-1**	**SPF-2**
04-05-2021	5.78	6.02	25.3	25.5
11-05-2021	5.61	5.92	25.9	25.6
18-05-2021	5.94	6.04	26.2	26.0
24-05-2021	6.01	5.93	26.8	27.0
01-06-2021	5.84	6.04	26.3	26.1
08-06-2021	6.12	6.08	25.4	25.1
15-06-2021	6.43	6.12	25.1	25.4
29-06-2021	6.08	6.44	25.6	25.9
01-07-2021	5.95	6.32	26.2	26.5
06-07-2021	5.66	5.87	27.1	27.4
13-07-2021	5.59	6.09	27.9	28.2
20-07-2021	5.14	-	26.9	-
27-07-2021	5.57	6.32	27.4	27.9
09-03-2022	5.71	5.83	28.1	27.7
15-03-2022	5.18	5.79	27.9	27.5
22-03-2022	5.38	5.84	28.1	27.8
12-04-2022	5.65	5.91	27.6	27.4
19-04-2022	5.17	5.82	27.9	27.7
26-04-2022	5.39	6.08	27.0	26.8
10-05-2022	-	6.14	-	26.1

- No sample

Table S2. Mean values of pH and temperature from the effluent samples collected from the two seafood processing facilities (SPF) in Tema, Ghana during 2021 and 2022.

Parameter	Mean	SD	Min	Max	Mann Whitney U test	p-value
pH	5.86	0.32	5.14	6.44	72	0.00161
Temperature(°C)	26.74	0.98	25.1	28.2	190.5	0.7812

The Mann Whitney U test was used to test for significant difference in pH and temperature of samples from SPF-1 and SPF-2.

The test statistic from the analysis revealed statistically significant difference in pH level between SPF-1 and SPF-2 (p-value = 0.0012). On the contrary, the analysis revealed no statistically significant difference in temperature levels between both sites (p-value = 0.7812).
