# Supplementary figures and images for: Surveillance of WHO Priority Gram-Negative Pathogenic Bacteria in Effluents from Two Seafood Processing Facilities in Tema, Ghana, 2021 and 2022: A Descriptive Study
Source: Int J Environ Res Public Health. 2022 Aug 30;19(17):10823. doi: 10.3390/ijerph191710823 (PMC9518130; doi:10.3390/ijerph191710823)

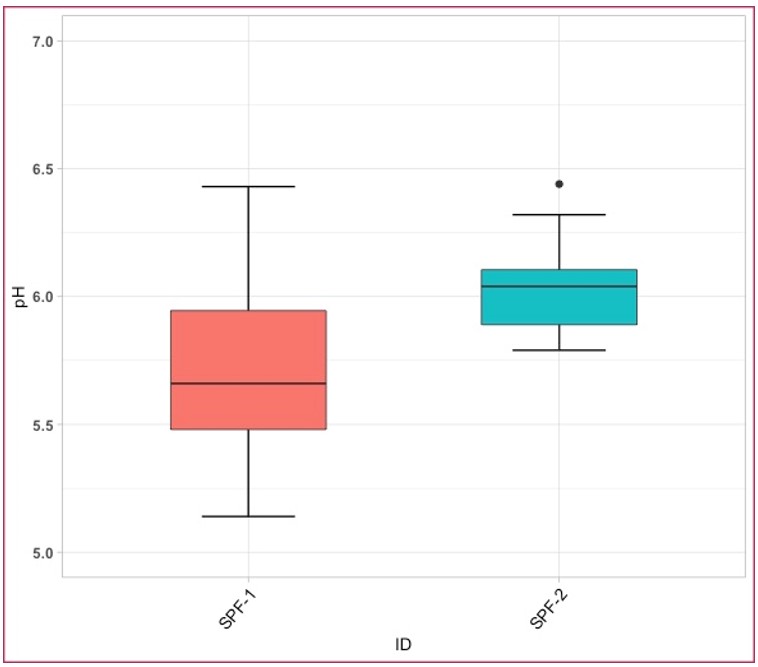

Supplement: Supplementary file 1 [file ijerph-19-10823-s001.zip › ijerph-1873579 Supplementary/Figure S1.jpg]

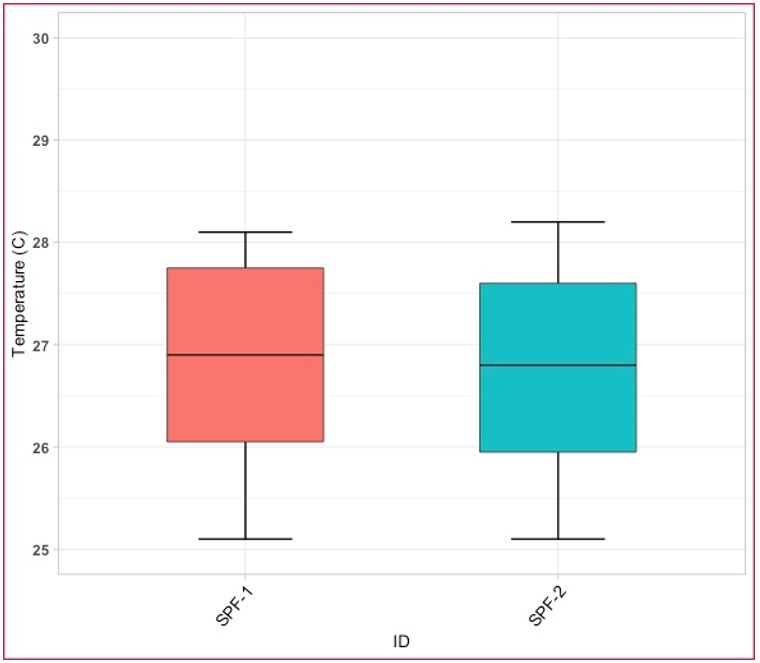

Supplement: Supplementary file 1 [file ijerph-19-10823-s001.zip › ijerph-1873579 Supplementary/Figure S2.jpg]
